# Potential Neuroimmune Interaction in Chronic Pain: A Review on Immune Cells in Peripheral and Central Sensitization

**DOI:** 10.3389/fpain.2022.946846

**Published:** 2022-07-04

**Authors:** Jia-Xuan Yang, Hong-Fei Wang, Ji-Zhun Chen, Han-Yu Li, Ji-Chen Hu, An-An Yu, Jun-Jun Wen, Si-Jia Chen, Wei-Dong Lai, Song Wang, Yan Jin, Jie Yu

**Affiliations:** ^1^Fourth School of Clinical Medicine, Zhejiang Chinese Medicine University, Hangzhou, China; ^2^First School of Clinical Medicine, Zhejiang Chinese Medicine University, Hangzhou, China; ^3^Second School of Clinical Medicine, Zhejiang Chinese Medicine University, Hangzhou, China; ^4^Institute of Clinical Fundamentals of Traditional Chinese Medicine, School of Basic Medicine, Zhejiang Chinese Medicine University, Hangzhou, China; ^5^Second Affiliated Hospital, Zhejiang Chinese Medical University, Hangzhou, China

**Keywords:** immune cells, chronic pain, peripheral sensitization, central sensitization, neuroimmune

## Abstract

Chronic pain is a long-standing unpleasant sensory and emotional feeling that has a tremendous impact on the physiological functions of the body, manifesting itself as a dysfunction of the nervous system, which can occur with peripheral and central sensitization. Many recent studies have shown that a variety of common immune cells in the immune system are involved in chronic pain by acting on the peripheral or central nervous system, especially in the autoimmune diseases. This article reviews the mechanisms of regulation of the sensory nervous system by neutrophils, macrophages, mast cells, B cells, T cells, and central glial cells. In addition, we discuss in more detail the influence of each immune cell on the initiation, maintenance, and resolution of chronic pain. Neutrophils, macrophages, and mast cells as intrinsic immune cells can induce the transition from acute to chronic pain and its maintenance; B cells and T cells as adaptive immune cells are mainly involved in the initiation of chronic pain, and T cells also contribute to the resolution of it; the role of glial cells in the nervous system can be extended to the beginning and end of chronic pain. This article aims to promote the understanding of the neuroimmune mechanisms of chronic pain, and to provide new therapeutic ideas and strategies for the control of chronic pain at the immune cellular level.

## Introduction

Chronic pain (CP) usually develops from the acute to a chronic state (1) and persists after expected healing, or exists in the absence of tissue damage, as a result of complex neural network interactions (2). Chronic pain, including neuropathic pain, chronic inflammatory pain, cancer pain, etc., has more complex clinical manifestations than acute pain. Neuropathic pain may be caused by disorders of various etiologies that affect the peripheral or central nervous system (e.g., diabetic neuropathy), and its diagnosis requires a history of injury or disease of the nervous system, and a reasonable neuroanatomical distribution of pain (3). Inflammatory pain usually is caused by pro-inflammatory mediators, characterized by sensitization both at the site of damage and in the adjacent area (4). Besides, the mechanisms of cancer pain include the release of inflammatory mediators induced by cancer tissue and the destruction of sensory nerves, resulting in neuropathy. In addition to unique characteristics, cancer pain also has a mix of inflammatory and neuropathic features (5).

Peripheral and central sensitization usually occurs during the development of chronic pain. Peripheral sensitization refers to increased sensitivity of peripheral afferent nerves (mainly Aδ and C fibers) to mechanical and thermal stimuli after nerve injury, and increased input of nociceptors, which is confined to the site of injury (6). Central sensitization, on the other hand, refers to a high degree of sensitivity of the central nervous system (CNS), due to persistent activation of peripheral nociceptive afferent nerves (especially C fibers) or exposure to repeated effects of noxious stimuli, as evidenced by the persistence of abnormal pain responded to non-noxious stimuli. Hypersensitivity of the nervous system is also the main cause of chronic pain that is unsatisfied with the control or relief of conventional analgesic drugs, which seriously affects the lives of patients.

**Figure 1:**
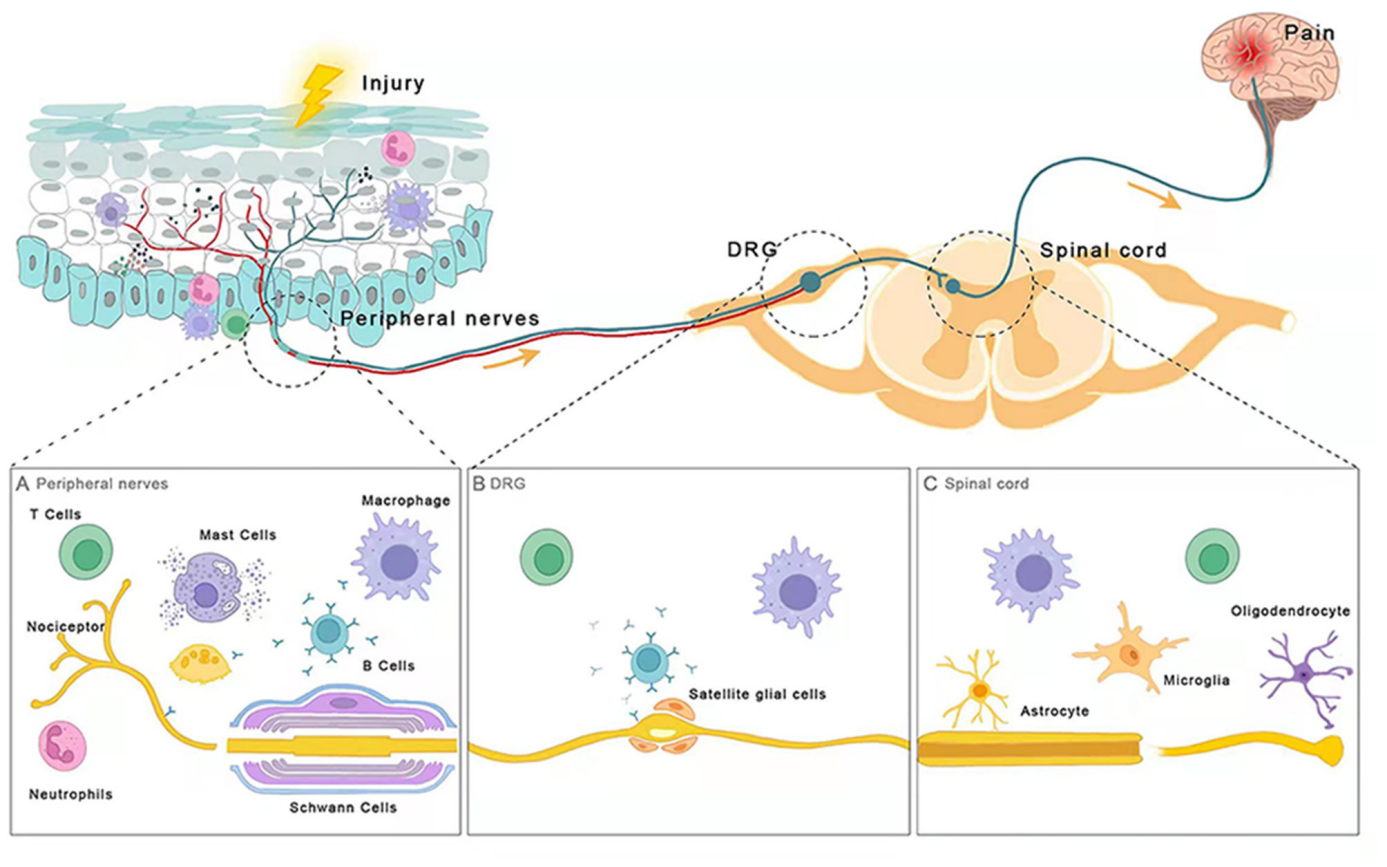
Immune cells are involved in chronic pain. **(A)** Peripheral nerves **(B)** DRG **(C)** Spinal cord. After injury, distinct parts of the nociceptive pathway interact with different types of immune cells including intrinsic immune cells (macrophages, neutrophils, and mast cells), adaptive immune cells (B and T lymphocytes), and glial cells (oligodendrocytes, and astrocytes, microglia). These immune cells release neuromodulators near nociceptors and sensory neurons to modulate their sensitivity and excitability, promoting or dampening chronic pain.

In recent years, many researchers have explored a lot in the interaction between the immune system and the sensory nervous system of chronic pain. Intrinsic immune cells (macrophages, neutrophils, and mast cells), adaptive immune cells (B and T lymphocytes), and central glial cells build the main immune defense that usually malfunctioned in patients with chronic pain. A growing number of studies have found that multiple immune cells are critical for the development of chronic pain, as well as its relief. The action of immune cells may facilitate or postpone the pain from acute to chronic state. For example, neutrophils, macrophages, and mast cells are recruited to relevant tissues in the early stages of inflammation and may initiate the transition from acute to chronic pain, as well as the subsequent maintenance and resolution of pain; B cells and T cells are mainly involved in the progression of chronic pain, and T cells also contribute to the resolution of chronic pain. Furthermore, central glial cells are found to be present throughout the development of chronic pain. Therefore, an in-depth discussion of the mechanisms by which these immune cells directly or indirectly regulate the sensitivity of the peripheral and central nervous system, and how they are involved in the initiation, maintenance, and resolution of chronic pain, would be extremely useful for understanding the pathological progression of chronic pain. In this review, we try to discuss the mechanisms by which immune cells cause sensitization of the peripheral sensory nervous system or partially migrate to the CNS to initiate central sensitization. We also speculated several potential targets for intervene of the chronic pain in clinic. It will provide new therapeutic ideas and broad clinical treatment prospects for chronic pain control, and offer more possibilities to improve the life quality of patients suffering from chronic pain (Figure 1).

## Immune Cell Participates in Peripheral Sensitization

### Neutrophils

Neutrophils are mainly polymorphonuclear leukocytes produced by myeloid precursor cells in the bone marrow. In the early stage of inflammation or injury, neutrophils are among the first immune cells initially recruited to locally injured tissues. Their migration and the release of inflammatory mediators play a leading role in early events of inflammation (7). Activated neutrophils can be involved in the transition from acute to chronic pain (8). Studies have found that neutrophils could release inflammatory mediators to sensitize nociceptors during the acute phase of nerve injury. Then, it recruited other immune cells such as macrophages and T cells to the injury site, causing them to secrete large amounts of pro-inflammatory factors to initiate and maintain pain (9, 10). In the chronic constriction injury (CCI) model, neutrophils migrate to the vicinity of the dorsal root ganglia (DRG) and express the chemokine MCP-1/CCL2, which in turn sensitizes peripheral nociceptors (11). PGE2 released by neutrophils can promote the occurrence of chronic pain by stimulating DRG neurons to release pain-related neuropeptides (e.g., SP and CGRP) through direct or indirect action (12). Interleukin (IL)-18 secreted by neutrophils also plays a central role in the maintenance of chronic pain. In the muscle pain model, the increasing number of neutrophils and ATP is continuously contracted muscle tissue and stimulates the production of IL-18, which in turn promotes neutrophil migration. Therefore, the number of neutrophils at the injury site would continue to grow, maintaining the development of pain (13). Furthermore, in the CCI model, IL-18 can feedback control its expression and regulate the other mediators involved in neuroinflammation, such as causing an up-regulation of MCP-1/CCL2, IL-4, or a down-regulation of IFN-γ (14). In patients with rheumatoid arthritis (RA), neutrophil extracellular traps (NETs) induced hyperalgesia by acting on Toll-like receptors (TLR-4 and TLR-9), and the production of NETs was positively correlated with the degree of pain (15). Other studies also reported that neutrophils infiltrated around the affected DRG release leukocyte elastase (LE) and played a role in the maintenance of chronic neuropathic pain in a diabetic neuropathic pain model. Therefore, blocking the site of action of LE in DRG and peripheral tissues with inhibitors may be more effective in the treatment of chronic pain (16).

Additionally, neutrophils also play a role in the relief of chronic pain. Neutrophils at the site of inflammation can secrete analgesic mediators (e.g., opioid peptides) to relieve pain (17). Besides, neutrophils suppress immune responses, such as inhibiting T cell responses through macrophage antigen-1 (Mac-1) (18), which may contribute to pain relief. There before, prophylactic inhibition of neutrophil immune response or blocking the transmission of immune mediator targets prevent the development of chronic pain, but some substances like NETs may play a dual role (19). The mechanisms of neutrophils affecting chronic pain still need further research.

### Macrophages

Macrophage (MΦ), derived from monocytes, is a type of leukocyte located in tissues. Their main function is to engulf cellular debris and pathogens, and at the same time, activate lymphocytes or other immune cells that are involved in innate and cellular immunity of vertebrates. An increased number of macrophages have been observed in tissues related to pain, such as skin, DRG, or injured nerves (20–24). These macrophages are often recruited and activated by chemokines released from activated neutrophils.

Macrophages in peripheral tissues participate in the initiation and maintenance of chronic pain by inducing the onset of peripheral sensitization (25). When infection, tissue injury, or chemotherapy occurs, macrophages are induced to produce damage-associated molecular pattern molecules (DAMP) and pathogen-associated molecular pattern molecules (PAMP). They activate TLRs expressed in nociceptive neurons and up-regulate the expression of myeloid differentiation factor 88 (MyD88), which promotes the mitogen-activated protein kinase (MAPK) signaling pathway at the injury receptor terminals via κ-gene binding nuclear factor (NF-κB). At the same time, MyD88 also activates sodium channels in sensory neurons, thus increasing the excitability of nociceptors (26, 27). Secondly, sensory neuron-associated macrophages (sNAMs) induce nociceptive neurons hypersensitivity by releasing pro-inflammatory mediators and recruiting other leukocytes (e.g., blood CCR2 monocytes). In addition, there is also aggregation and activation of sNAMs in the DRG after peripheral nerve injury. These macrophages can mediate the development of neuropathic pain by initiating cytokines (e.g., IL-1β) and reactive oxygen species (ROS) (28).

**Figure 2:**
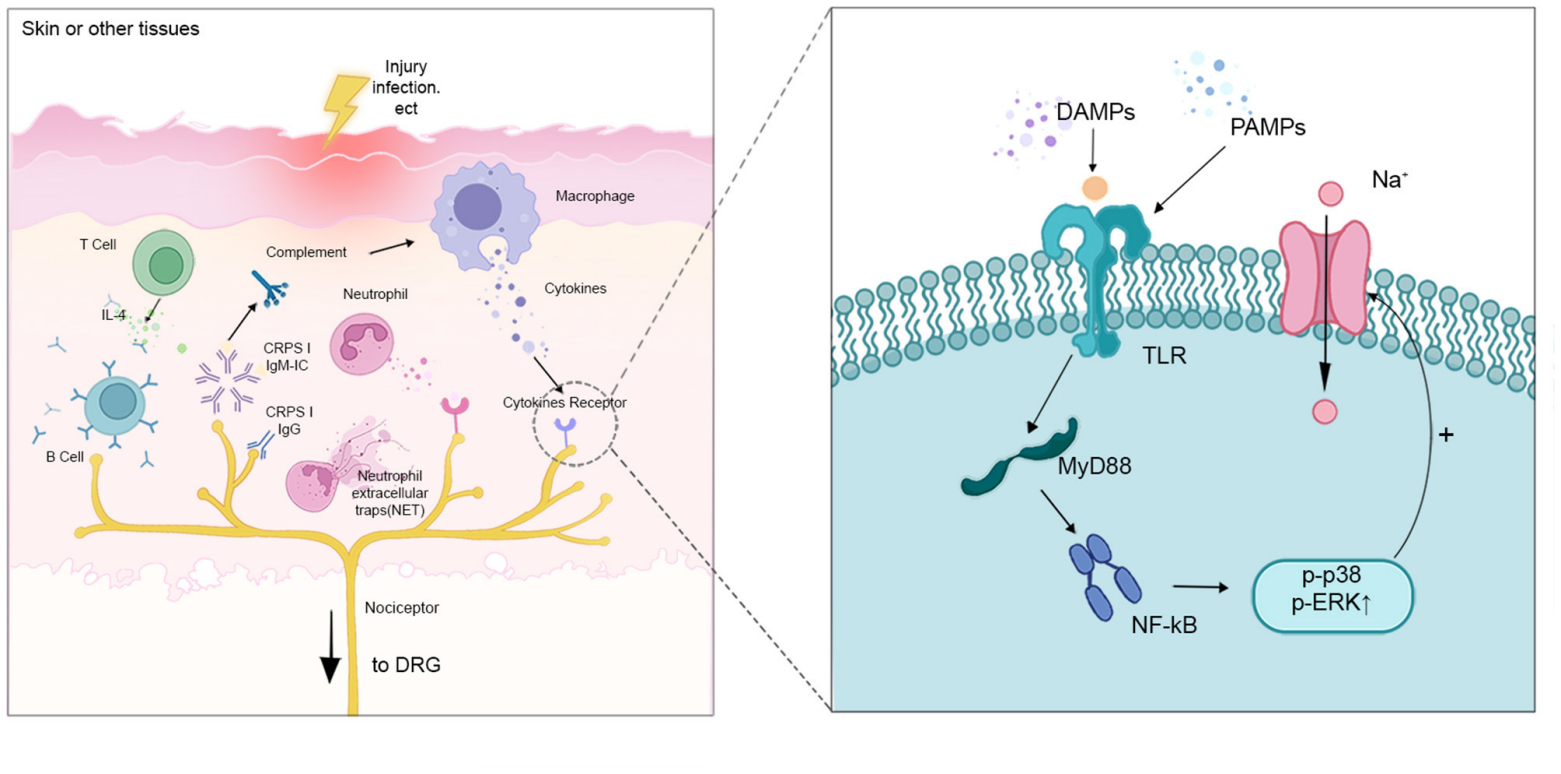
Interactions between the distal part of nociceptors with different immune cells. When injury and infection happen, macrophages are induced to produce DAMP and PAMP. They activate TLRs expressed in nociceptive neurons and up-regulate the expression of MyD88, which promotes the MAPK signaling pathway through NF-κB. MyD88 also activates sodium channels in sensory neurons, increasing the excitability of the nociceptors. T cells release IL-4 to activate B cells, which produce CRPSI IgM and IgG. CRPS IgM, binding to the corresponding neoantigens. The antigen-IgM complex promotes complements (e.g., C5a), leading to increased expression of pro-inflammatory cytokines and peripheral sensitization.

Macrophages also play a role in alleviating chronic pain and promoting the resolution of pain. Studies have shown that local M2 macrophages released IL-4, directly inducing sustained initiation of opioid peptides and ameliorating pain (29). IL-10, which is also produced by M2 macrophages, is essential for the relief of transient nociceptive hypersensitivity. So, it is unclear whether the low expression of GRK2 or down-regulation of IL-10 is peripheral biomarkers of the risk of chronic pain after inflammation, and future studies are needed to clarify this issue (30). In addition, studies have shown that macrophages could prevent the transition from acute to persistent inflammatory pain (31). M2 macrophages also transfer mitochondria to sensory neurons in the DRG and restore their oxidative phosphorylation to reduce the excitability of sensory neurons, contributing to the resolution of inflammatory pain as well (32) (Figure 2).

### Mast Cells

Mast cells, inducing an innate immune response mainly at an early stage, play a role in anti-infection and pain induction by being recruited and degranulation. The high-affinity IgE receptor (FcεRI) on the surface of mast cells binds to IgE and activates the secretion of intracellular pro-inflammatory mediators (33), a degranulation process that promotes inflammation. Mast cells also degranulate in response to immunostimulation and non-immunostimulation (e.g., stress) (34). They release histamine, bradykinin, cytokines, chemokines, lipids, and other inflammatory mediators, leading to up-regulated sensitivity of peripheral pain-related neurons (Aδ and C fibers, etc.), which cause hyperalgesia in chronic pain.

Histamine released by mast cells plays a dominant role in the development of peripheral sensitization. It has been shown to increase the mechanical sensitivity of peripheral neurons through the activation of histamine H1 receptors and capsaicin receptors (TRPV1). Histamine can also induce neutrophil and monocyte recruitment to promote the development of hyperalgesia (35). Mast cell stabilizers or pre-administration of histamine receptor antagonists can effectively relieve chronic pain (36, 37). However, symptoms of chronic pain did not completely disappear after administration of histamine receptor antagonists, suggesting that other inflammatory mediators (e.g., serotonin, bradykinin) released by mast cells may also be involved in the development of chronic pain (38). Furthermore, leukotrienes released by mast cells can recruit neutrophils and promote the release of pro-inflammatory mediators (39) and trypsin sensitizes the TRPV1 by activating protease-activated receptor 2 (PAR-2) to sensitize the periphery. TNF-α released from mast cells causes a decrease in the stimulation threshold and induces abnormal firing of dorsal root neurons (40). In another hand, mast cells can affect the nociceptive transmission, increasing the excitability of C-fibers. Mast cell-dependent cell adhesion molecule 1 (CADM1) and other substances can also form mast cell nerve synapses with nerve synapses (41), making mast cells more sensitive to signals released by neurons. Recent studies reported that immune cells interact with each other through inflammatory mediators, such as TNF-α released by monocytes/macrophages affecting the release of TNF-α from mast cells (42). In addition to inflammatory mediators, FcεRI levels in mast cells are reduced by activation of Treg cells, thus preventing mast cell degranulation (43).

Thus, there are synergistic or antagonistic effects between mast cells and other immune cells, which makes the intervene unpredictable, and the effectiveness for the treatment of chronic pain needs to be further studied and proven. Additionally, future studies can also be conducted to investigate effect of regulation of mast cells on the growth of pain-related neurons, either in the peripheral or the central nerve system.

### B Lymphocytes

B lymphocytes not only perform specific humoral immune functions by producing antibodies but also present antigens to T cells, and participate in immune regulation by secreting cytokines. Numerous studies have suggested that pathogenic B cells may influence the development of chronic pain. For example, several controlled clinical trials have shown that B-cell-targeted therapy with rituximab is effective in patients with rheumatoid arthritis (RA) and significantly alleviates their pain (44, 45).

B lymphocytes play a role in peripheral sensitization primarily through the secretion of autoantibodies. Intravenous injection of purified anti-citrullinated protein antibodies (ACPA) from RA patients or arthritic mice into healthy mice was found to cause persistent pain and increase their nociceptive sensitivity. This is because ACPA mediates the expression and release of chemokine CXCL1 (like human IL-8) by osteoblasts, which in turn increases the sensitivity and excitability of nociceptive neurons via the receptor CCR2, without causing inflammation (46). Furthermore, in an animal model of complex regional pain syndrome type I (CRPSI), an autoimmune disease associated with chronic post-traumatic pain, serum IgM from fractured WT mice had a pronociceptive effect on the fractured limb of μMT mice lacking functional B cells (47). This may be due to the binding of CRPSI IgM to neoantigens in the skin of the fractured limb, and the antigen-IgM complex subsequently promotes C5a signaling in skin macrophages, leading to increased expression of pro-inflammatory cytokines at the corresponding sites, resulting in increased sensitivity of skin nociceptive neurons (48). It has also been shown that CRPSI IgG perpetuates nociceptive hypersensitivity in chronic CRPSI through sensitization of A and C fibers (49). The associated autoantibodies in patients with RA can be positive even years before the clinical onset of the disease, which may be closely related to the early presence of pain before the onset of clinical symptoms in patients with RA (50).

In addition to acting indirectly on neurons by mediating the release of cytokines from other immune cells, autoantibodies can directly lead to increased neuronal excitability. The voltage-gated potassium channel (VGKC) is one of the targets of autoimmunity in chronic pain. Antibodies to contactin-associated protein-like 2 (CASPR2-Ab) in the VGKC complex cause a decrease in the expression of the VGKC Kv1 subunit on the surface of the DRG and an increased mechanosensitivity of Aδ fibers, which disrupt normal hyperpolarizing currents and lead to increased excitation of the action potential, resulting in impaired repolarization and consequent hyperexcitability of nociceptive neurons (51, 52). Furthermore, functional FcγRI is expressed in dorsal root ganglion cells and subpopulations of nociceptive neurons. Activation of this receptor by IgG immune complexes (IgG-IC) increases neuronal excitability and leads to up-regulation of FcγRI expression itself (53, 54). Increased excitability of neurons is mediated primarily by FcγRI, which causes intracellular calcium release via spleen tyrosine kinase (Syk), triggering the opening of the classical transient receptor potential channel 3 (TRPC3), leading to increased depolarization and firing in DRG nociceptive neurons (53, 54). Whether other types of FcγR participate in this process remains to be further investigated.

It strongly suggests the role of autoantibodies from B lymphocytes as an important neuroimmune regulated target in arthritic chronic pain. However, it remains unclear what causes the initial transition from immune tolerance to autoimmune activation, and triggers autoantibody production from autoantigen-specific B cells. This is an unresolved issue with significant clinical implications for the prevention and treatment of arthritic chronic pain.

### T Lymphocytes

T lymphocytes are derived from lymphoid progenitor cells in the bone marrow, which are key components of the adaptive immune system, with roles in immune memory and regulation of other immune cell activities. Currently, existing evidence has indicated that T cells not only play a key role in the initiation of chronic pain but also contribute to the resolution of pain.

In peripheral nervous systems, T cells can be directly regulated by sensory neurons of the dorsal root and trigeminal ganglion. Local T cell immune activity is affected by the release of neurotransmitters (e.g., glutamate) and neuropeptides (e.g., calcitonin gene-related peptides) from sensory neurons (55). Several studies have found that, during nerve injury, dendritic cells activate T cells through antigen presentation and promote T cell differentiation into Th1 cells. Th1 cells accumulate and infiltrate the site of injury and the distal end of the nerve, then the DRG, where IFN-γ is secreted by Th1 cells. Finally, IFN-γ activates glial cells and initiates chronic pain (10, 56).

The CNS will induce the release of Ach from the vagus nerves through multiple pathways. After receiving signals such as inflammatory injury in the periphery, it activates Ach receptors of immune cells and regulates their production of the corresponding cytokines to modulate the peripheral inflammatory response. For example, T cells are regulated by the sympathetic and vagal tone, and release Ach to inhibit the production of inflammatory mediators from macrophages, thus achieving anti-inflammatory effects (57). As mentioned above, M2 macrophages play an important role in the resolution of chronic pain. T cells that have been activated can produce the corresponding cytokines (e.g., IL-10) to promote the differentiation of macrophages into the M2 subpopulation, indirectly alleviating chronic pain (58).

In addition, T cells also have a direct effect on alleviating chronic pain. It is likely that CD4^+^ T cells produce IL-10. Furthermore, similar to macrophages that can produce endogenous opioid peptides in pain models, some CD4^+^ T cells were found to suppress pain in models of inflammatory bowel disorder through the release of enkephalins (59). A comparative study between control mice and T-cell-deficient mice revealed that endorphin T-cell production is regulated by exogenous opioid peptides. It indicates that T cells may also play a role in analgesic effects when opioids are provided from an exogenous source (60, 61).

**Figure 3:**
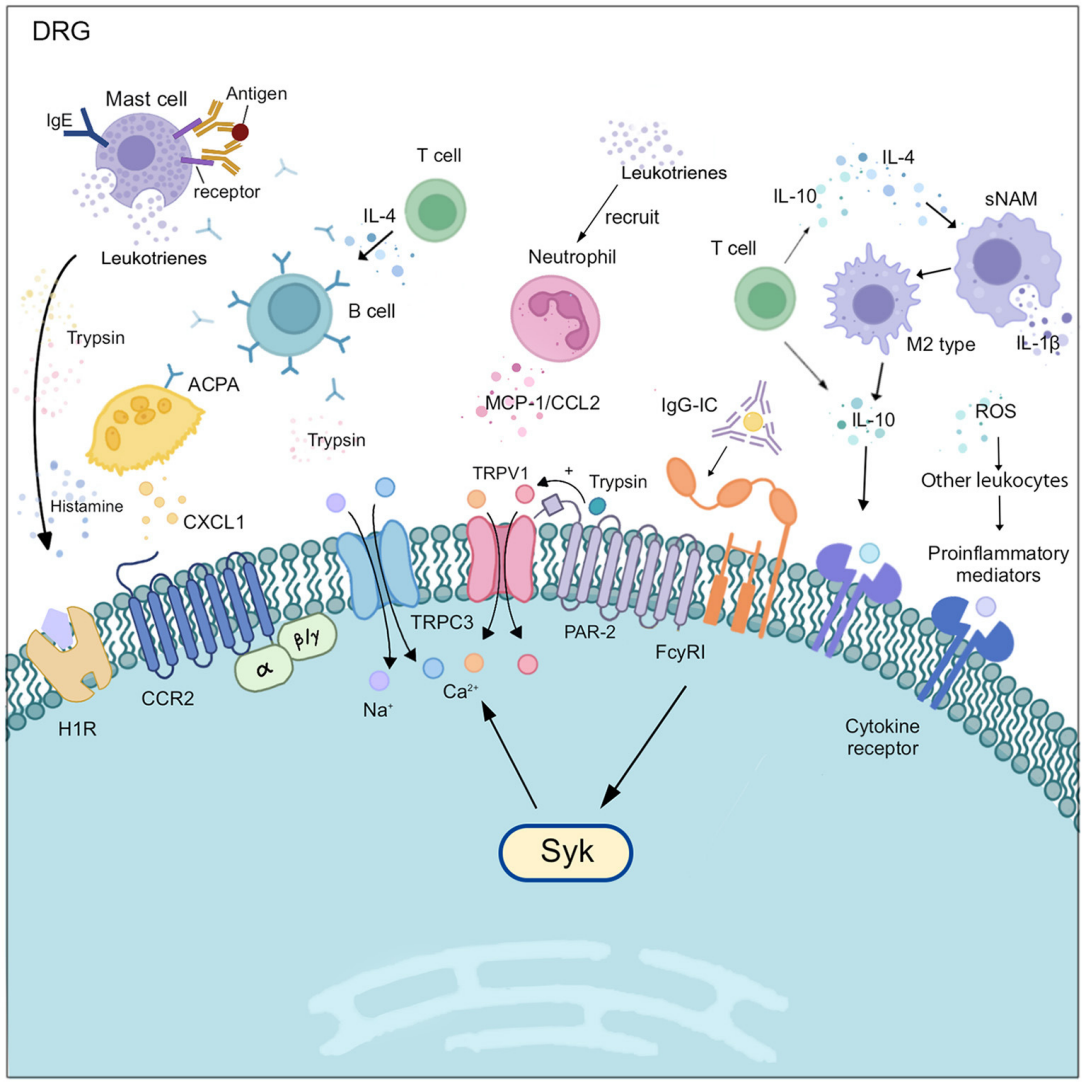
Various immune cells interact with a nociceptive neuron in DRG. In DRG, macrophages, neutrophils, and mast cells secret corresponding cytokines to activate the excitability of sensory neurons and recruit more leukocytes. B cells produce antibodies and initiate consequent activation of nociceptive neurons. Meanwhile, T cells modulate B cells and macrophages through IL-4 or IL-10, and also interact with nociceptive neurons.

In healthy conditions, the distribution of T cell subsets is mostly constant in peripheral tissues. However, several studies of different chronic pain-related diseases have found that there may be a degree of variability between the phenotypes of T lymphocyte subsets (62, 63). For example, CD4^+^/CD8^+^T lymphocytes were significantly elevated in patients with regional pain, such as osteoarthritis and chronic low back pain, while there was a significant decrease trend in CD39^+^ lymphocyte-specific subpopulations (64–66). It was found that the subsets of T cells that suppress pain varied in different models of chronic pain and played a regulatory role in pain resolution. However, future research is recommended to elucidate the specific mechanism, by which they promoted pain resolution (67). Significantly, external factors such as smoking and pain medication, as well as age and gender, will influence the development of T cell phenotypes. The study of the differences and common characteristics of T cell subpopulations is expected to be crucial in the search of new targets for the treatment of arthritic chronic pain (Figure 3).

## Immune Cell Involves in Central Sensitization

### Central Glial Cells

Microglia, erythroid-like progenitor cells originating from the yolk sac, are macrophages of the CNS and play an active role in regulating homeostasis and maintaining normal physiological conditions in the CNS. In response to peripheral nerve injury or inflammation, microglia will be activated by signaling factors such as ATP, colony-stimulating factor-1 (CSF1), chemokines (e.g., CCL2, CX3CL1) and proteases secreted by neurons, T lymphocytes, or other cells. Then, cell body enlargement, morphological changes, and an increase in cell surface chemotactic receptors will occur (68). Furthermore, a series of biochemical changes occur, such as increased secretion of cellular markers (e.g., IBA-1 and CD11b) (69), phosphorylation of the p38 mitogen-activated protein kinase (70), and up-regulated expression of the ATP receptor P2X_4_ and the chemokine receptor CX3CR1 (71). These reactions eventually lead to increased production and release of neuromodulators such as TNF-α, IL-1β, brain-derived growth factor (BDNF), cyclooxygenase (COX), and prostaglandin E2 (72). These neuromodulators directly or indirectly increase the excitability of neurons involved in nociceptive transmission pathways and induce central sensitization to chronic pain. In addition, microglia are also involved in the maintenance of chronic pain. Activation of microglia disrupted chloride homeostasis outside neurons, which is associated with opioid-induced nociceptive sensitization (73). Minocycline, a non-selective microglia inhibitor, has been shown to reduce neuropathic, inflammatory, and postoperative pain, but its analgesic effect in reducing established, late-onset neuropathic pain is limited (74). In a mouse model of morphine-induced pain sensitization, chronic pain caused by central sensitization can be effectively relieved by selective inhibition of spinal microglia.

Chronic pain caused by peripheral nerve injury could also results in activation of microglia in the limbic reward circuit, leading to disruption of dopaminergic signaling and reward behaviors in the midbrain (75). This suggests that chronic pain has an effect on areas of the central nervous system that is important for mood and emotion, making chronic pain associated with anxiety and depression. Besides, the P2Y12 receptor (P2Y12R), a metabolic purinoceptor that is expressed on microglia in the brain, has been indicated to play a critical role in the pathogenesis of chronic pain (76).

As the most abundant cell in the CNS, astrocytes play a crucial role in maintaining homeostasis in the brain and spinal cord. For example, it participates in neurotransmitter recycling, blood-brain barrier formation, regulation of extracellular ion concentration, and synaptic transmission. Similar to hyperreaction of microglia, astrocytes will undergo significant changes in phenotype, function, and gene expression upon activation by relevant signaling factors, in response to peripheral stimuli (e.g., nerve damage). Reactive astrogliosis is a defensive response to the initial injury, such as increasing neuroprotection and nutritional support for insulin-stressed neurons, reconstructing the damaged blood-brain barrier, and limiting the infiltration of peripheral leukocytes (77). However, astrocyte activation not only directly or indirectly causes high expression of inflammatory factors such as IL-1β, TNF-α, and IL-6, which promote the development of chronic pain (78) but also loses the ability to maintain steady-state concentrations of extracellular potassium (K^+^) and glutamate, leading to hyperexcitability of neurons (79). Furthermore, it reported that the interaction between CXCL2 secreted by astrocytes and CXCR2 expressed by spinal cord neurons also contributes to neuropathic pain (80). CXCL13 expression in spinal cord neurons is upregulated after nerve injury and maintains neuropathic pain through the activation of CCR5 astrocytes (81). All these studies demonstrated the involvement of astrocytes in the development and maintenance of chronic pain. Intrathecal injection of astrocyte inhibitors, such as glutamate and l-1-aminoadipic acid, effectively reversed abnormal pain in pathological pain models (82). However, given its limited clinical samples, the fact that the key mechanisms of action in the CNS, its efficacy, and adverse effects in clinical practice are not fully understood, thus further research is urgently needed to target glial cells for the treatment of chronic pain.

In recent years, a growing number of studies have demonstrated the involvement of glial cells in the development and maintenance of chronic pain, and the feasibility of drug inhibition of glial cells as the treatment. However, most of the related drugs have not entered the stage of clinical trials and their efficacy and side effects have not been clearly defined, requiring further research. Additionally, most studies have focused on a single signaling pathway involving microglia or astrocytes in the development of chronic pain. Although a few studies have confirmed that interactions between glial cells and signaling pathways are also involved in chronic pain, further studies are still needed.

### Peripheral Immune Cells Participate in Central Sensitization

Neutrophils play a role in linking peripheral inflammation with immune activation in different regions of the CNS, especially the prefrontal cortex (PFCTX). During persistent inflammatory pain, up-regulation of immune genes, such as S100A8, S100A9, and LCN2 in the PFCTX, lead to neutrophil infiltration and the increase of PFCTX neuronal activity (83). Certain genes, such as S100A8 and S100A9, were only upregulated in the PFCTX, indicating the site-specific changes of neuroimmune interaction in CNS may be potential targets for pain control.

Specific environments and stimuli have a significant effect on the number and state of mast cells, making mast cells involved in the initiation of chronic pain. It was reported that small amounts of mast cells are in the normal brain (34). These mast cells release many types of pro-inflammatory mediators through degranulation processes, causing central sensitization. For example, histamine, a pro-inflammatory mediator released by mast cells, can also increase the number and sensitivity of activated spinal cord dorsal horn neurons through receptors (84). In chronic migraine, a mast-cell-specific receptor MRGPRX2 has also been shown to be activated by a neuropeptide (SP) in the brain (85), which induces mast cell degranulation (86). Meanwhile, central sensory neurons continue to release SP under positive feedback regulation, resulting in additional activation of mast cells. Another mast-cell-specific receptor, MRGPRB2, is also activated by SP and acts to recruit immune cells.

Due to the presence of an intact blood-brain barrier and blood-spinal cord barrier, peripheral immune cells cannot enter the brain parenchyma and spinal cord. Only small amounts of cells (e.g., T cells) and inflammatory mediators slowly enter the surrounding meninges through the meningeal lymphatics (87). However, recent research has found that both nerve injury and peripheral inflammation cause increased permeability of these blood-central nervous system barriers. For example, endothelin-1 produced by microglia and astrocytes after nerve injury can increase the permeability of the blood-central barrier, while these two cells also secrete a variety of chemokines and cytokines that promote peripheral leukocytes (including macrophages and T cells, etc.) to adhere to and cross the endothelium of the blood-central barrier (88–90). In addition to increased activation of the MAPK signaling pathway in spinal microglia, proliferation and activation of both microglia and astrocytes are jointly involved in the initiation of neuropathic pain (91). Furthermore, the ATP-sensitive P2X_7_ receptor on peripheral monocytes/macrophages and central glial cells plays a specific role via regulation of mature IL-1β production and neural-glial cell interactions in the development of chronic pain (92, 93). Activation of T cells entering the CNS produces corresponding cytokines that affect central sensitization by activating or inhibiting central glial cell responses. It has been found that CD4^+^ αβ T cells selectively infiltrate the soft meninges after peripheral nerve injury. Their production of IFN-γ not only induces glial cell activation in the spinal cord but also participates in the transition from acute to chronic pain after nerve injury (94). With peripheral tissue inflammation, T cells (mainly Th1) migrate to the spinal cord and release IFN-γ, which activates astrocytes and induces chronic pain (95). On the other hand, IL-10 and opioid peptides produced by T cells and M2 macrophages reverse the sensitization of neurons in the CNS and inhibit microglial activity (59, 96). In summary, IFN-γ released from T cells activates astrocytes, which in turn influence T cell activation and expression of related cytokines. A vicious cycle is formed between them, and the specific molecular mechanism of which needs further research. Interrupting this vicious cycle may be a target for treating pain without affecting the immune function of T cells themselves. T cells in the meninges secrete IL-4 to trigger the production of brain-derived neurotrophic factors, and neurogenesis occurs in the brain (97). Studies have shown that IFN-γ derived from meningeal T cells also plays a role in antigen-antibody reaction in CNS, and regulates specific neuronal circuits in the steady-state of meninges (98). Under pathological conditions, T cell could further enter the cerebrospinal fluid, directly regulating the activity of neurons in the CNS (98). Therefore, the study of the mechanism by which immune cells passing through the meningeal barrier is of great significance.

Under pathological conditions, B cells can be activated by locally produced cytokines and enter the CNS too (99). In multiple sclerosis, B cells secret various cytokines (GM-CSF, IL-6, IL-10, etc.), which activate different immune cells (e.g., microglia and T cells) and influence the inflammatory environment of the CNS (100–102). IL-6 secreted by B cells may have a remarkable significance for maintaining neuropathic pain. This may be related to the fact that IL-6 promotes Th17 polarization, opening the blood-brain barrier and creating an environment for the persistence of B cells in the CNS (103).

**Figure 4:**
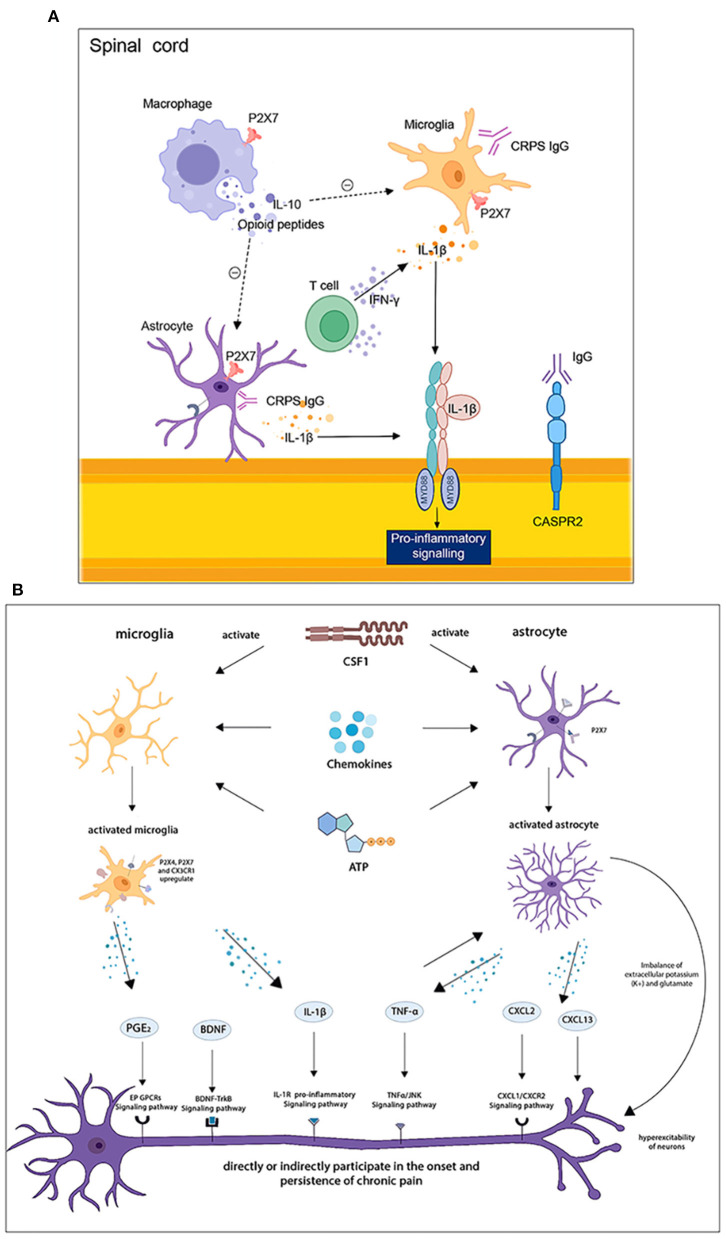
**(A)** Immune cells interplay in the spinal cord. **(B)** Central neuron-glial cells interactions in the spinal cord and brain. T cells release IFN-γ to activate astrocytes and induce pain, while macrophages produce substances such as IL-10 that promote pain resolution. Activated astrocytes and microglia secrete IL-1β, which promotes the release of pro-inflammatory factors. Astrocytes and microglia are activated by CSF1, chemokines, and ATP, and secrete BDNF, IL-1β, TNF-α, CXCL2, and other neuronal modulators, which are involved in the development and maintenance of chronic pain through corresponding signaling pathways.

Autoantibodies secreted by pathogenic B cells can also lead to central sensitization. As mentioned above, in addition to binding to neoantigens in the skin of the fractured limb, CRPS IgM binds to neoantigens in the corresponding spinal cord, and the antigen-IgM complex also promotes C5a complement signaling in spinal microglia, causing increased expression of spinal pro-inflammatory cytokines, and resulting in central sensitization (48). Other studies have also identified chronic CRPSI IgG as a major cause of chronic pain in patients with CRPSI. Serum IgG, transferred from chronic CRPSI patients to mice of the hind paw incision model, has been reported to significantly increase swelling of the incised paw and induce stable nociceptive hypersensitivity. It may be attributed to the fact that CRPSI IgG promotes microglia and astrocyte activation in the CNS, promoting central sensitization by up-regulating the secretion of IL-1β (104). Furthermore, the phenomenon that CASPR2-Ab induces pain-related hypersensitivity in mice may also be associated with interference of hyperexcitability at the spinal cord level of mice (51). As studies unveiled, autoantibodies also significantly account for arthritic chronic pain, but their mechanisms are less well-established compared to studies of cytokines. Thus, researchers should pay more attention to the role of autoantibodies in chronic pain (Figure 4).

## Conclusion

There has been growing evidence that immune cells influence chronic pain through their involvement in peripheral sensitization and central sensitization. First, immune cells originally in the periphery act on peripheral nociceptive sensory neurons to elevate their excitability directly or indirectly [by mediating the release of mediators from other immune cells, such as CXCL1 (46), IL-10 (105), IL-1β (78, 106), IL-6 (107)] (Table 1). On the other hand, some immune cells migrate into the CNS and participate in central sensitization together with glial cells in response to various stimuli. For example, neutrophils can synergistically cause chronic pain by releasing inflammatory mediators and recruiting macrophages and T cells (9, 10). Neutrophils can also induce nociceptive sensitization by releasing inflammatory mediators, such as histamine and leukotrienes. Mast cells also contribute to the recruitment of neutrophils and macrophages (35, 39); T cells induce the polarization of macrophages to the anti-inflammatory M2 type to relieve chronic pain (58). Additionally, the immune system and the nervous system bidirectionally interact with each other. Not only are mast cells and T cells regulated by neurons, but recent studies have also revealed the presence of sensory neurons around lymph nodes that may contribute differentially to immune responses by participating in local tissue-specific neuroimmune circuits (108). To gain a deeper understanding of the mechanisms underlying the development of chronic pain, the effects of the nervous system on immune cells in chronic pain states deserve further investigation.

**Table 1:** Relative cytokines of immune cells in the central and peripheral sensitization.

**Cell type**	**Related cytokines or mediators**
Neutrophils	MCP-1/CCL2 (11), PGE2 (12), IL-18 (13, 14), NETs (15), LE (16), opioid peptides (17)
Macrophages	MyD88 (26), IL-1β (25, 26, 29–31), IL-4 (26, 29, 109), IL-10(25, 26, 28–31, 109, 110), opioid peptides (29)
Mast cells	TNF-α (40, 42), Histamine (35), Leukotriene (39), Serotonin, Bradykinin (38)
B lymphocytes	ACPA (46), CRPSI IgM (47, 48), CRPSI IgG (49, 104), CASPR2-Ab (51, 52), GM-CSF (100), IL-6 (101), IL-10 (102)
T lymphocytes	IFN-γ (10, 94, 95), Ach (57), IL-10 (58, 59, 96), enkephalins (59), opioid peptides (59, 96)
Astrocytes	IL-1β, TNF-α, IL-6 (111), CXCL13 (81)
microglia	TNF-α, IL-6, IL-1β (111), COX, PGE2 (112), BDNF (72)

In summary, targeting modulation of specific immune cell phenotypes, cytokines, or lipid mediators holds considerable promise as a powerful approach for the targeted treatment of chronic pain-related disorders. By inhibiting the release of pro-inflammatory factors or stimulating the activation of analgesic cytokine signaling, it will be a new strategy to effectively alleviate chronic pain. It is also important to note that pain treatment by depleting certain immune cells should consider side effects such as immunosuppression. Overall, in-depth research and elucidation of the specific mechanisms by which immune cells affect the sensory nervous system through various pathways will have profound implications for the identification of new drug targets, the development of novel analgesic drugs, and improving patients' quality of life with chronic pain-related disorders.

## Author Contributions

J-XY, H-FW, J-ZC, H-YL, J-CH, and A-AY conceived and wrote this article. J-JW, S-JC, W-DL, and SW collected and analyzed the papers. YJ and JY revised and wrote the article. All authors approved the submitted version.

## Funding

This study was supported by National Natural Science Foundation (No. 81971052), National key Research and Development Program (No. 2018YFC1705500), Research Project of Zhejiang Chinese Medical University (2022JKZKTS04) and Zhejiang Provincial Natural Science Foundation of China (LY22H280008).

## Conflict of Interest

The authors declare that the research was conducted in the absence of any commercial or financial relationships that could be construed as a potential conflict of interest.

## Publisher's Note

All claims expressed in this article are solely those of the authors and do not necessarily represent those of their affiliated organizations, or those of the publisher, the editors and the reviewers. Any product that may be evaluated in this article, or claim that may be made by its manufacturer, is not guaranteed or endorsed by the publisher.
